# Preexisting Comorbidities Predicting COVID-19 and Mortality in the UK Biobank Community Cohort

**DOI:** 10.1093/gerona/glaa183

**Published:** 2020-07-20

**Authors:** Janice L Atkins, Jane A H Masoli, Joao Delgado, Luke C Pilling, Chia-Ling Kuo, George A Kuchel, David Melzer

**Affiliations:** 1 Epidemiology and Public Health Group, University of Exeter Medical School, UK; 2 Department of Healthcare for Older People, Royal Devon and Exeter Hospital, UK; 3 Center on Aging, University of Connecticut Health Center, Farmington

**Keywords:** COVID-19, Epidemiology, Morbidity, Mortality

## Abstract

**Background:**

Hospitalized COVID-19 patients tend to be older and frequently have hypertension, diabetes, or coronary heart disease, but whether these comorbidities are true risk factors (ie, more common than in the general older population) is unclear. We estimated associations between preexisting diagnoses and hospitalized COVID-19 alone or with mortality, in a large community cohort.

**Methods:**

UK Biobank (England) participants with baseline assessment 2006–2010, followed in hospital discharge records to 2017 and death records to 2020. Demographic and preexisting common diagnoses association tested with hospitalized laboratory-confirmed COVID-19 (March 16 to April 26, 2020), alone or with mortality, in logistic models.

**Results:**

Of 269 070 participants aged older than 65, 507 (0.2%) became COVID-19 hospital inpatients, of which 141 (27.8%) died. Common comorbidities in hospitalized inpatients were hypertension (59.6%), history of fall or fragility fractures (29.4%), coronary heart disease (21.5%), type 2 diabetes (type 2, 19. 9%), and asthma (17.6%). However, in models adjusted for comorbidities, age group, sex, ethnicity, and education, preexisting diagnoses of dementia, type 2 diabetes, chronic obstructive pulmonary disease, pneumonia, depression, atrial fibrillation, and hypertension emerged as independent risk factors for COVID-19 hospitalization, the first 5 remaining statistically significant for related mortality. Chronic kidney disease and asthma were risk factors for COVID-19 hospitalization in women but not men.

**Conclusions:**

There are specific high-risk preexisting comorbidities for COVID-19 hospitalization and related deaths in community-based older men and women. These results do not support simple age-based targeting of the older population to prevent severe COVID-19 infections.

The 2019 novel coronavirus (SARS-CoV-19) (1) presents with a wide spectrum of clinical disease presentations, from asymptomatic infection to respiratory failure with high mortality (2). However, little is known about what predicts hospitalization or mortality with COVID-19 in different individuals.

The majority of patients hospitalized with COVID-19 are older and have underlying medical conditions (3,4), with increased age being associated with clinical severity (5,6), including case fatality (4,7). The most frequent comorbidities reported in Chinese COVID-19 patient cohorts were hypertension (21.1%, 95% confidence interval [CI]: 13.0%–27.2%), diabetes (9.7%, 95% CI: 7.2%–12.2%), cardiovascular disease (8.4%, 95% CI: 3.8%–13.8%), and respiratory system disease (1.5%, 95% CI: 0.9%–2.1%) (8), whereas in a large US cohort common comorbidities were hypertension (56.6%), obesity (41.7%), and diabetes (33.8%) (6). Data on common comorbidities in hospitalized patients are important for understanding the acute treatment challenge, but it is unclear whether these conditions are common in COVID-19 hospital inpatients merely because they are also common in the older population. To identify those living in the community who are at most risk of hospitalization with COVID-19 (alone or with mortality), data are needed on which preexisting conditions are disproportionately common in such inpatients compared to the background population.

UK Biobank (UKB) is a community-based cohort of 500 000 participants currently aged 48–86 (9). Electronic linkage between UKB records and National Health Service COVID-19 laboratory test results in England are available from March 16 to April 26, 2020, thus including the peak of daily COVID-19 laboratory-confirmed cases in the current outbreak (10). During this period, testing of older groups was largely restricted to hospital inpatients with clinical signs of infection (11), so test positivity is considered a good marker of severe COVID-19 (12). Given the scarcity of cohort data on risk factors for hospitalized COVID-19 in older groups, including those who died, we analyzed baseline (2006–2010) demographic characteristics and preexisting diagnoses during UKB follow-up. Identifying the specific risk factors explaining why some older people in the community were hospitalized with COVID-19 (of whom a subset died) may give clues to underlying vulnerabilities and are critical to developing outbreak control policies that focus on individual risk and avoid imposing crude age-based public health controls (13).

## Method

### UKB Cohort

The UKB consists of more than 500 000 community volunteers aged 40–70 years at baseline (2006–2010), living close to 22 assessment centers in England, Scotland, and Wales (9). Baseline assessments included demographics, lifestyle, and disease history, with linkages to electronic medical records. UK Biobank ethical approval was from the North West Multi-centre Research Ethics Committee. The current analysis was approved under the UKB application 14631 (PI, D.M.).

### COVID-19 Test Status and Sample Selection

**Figure 1.**
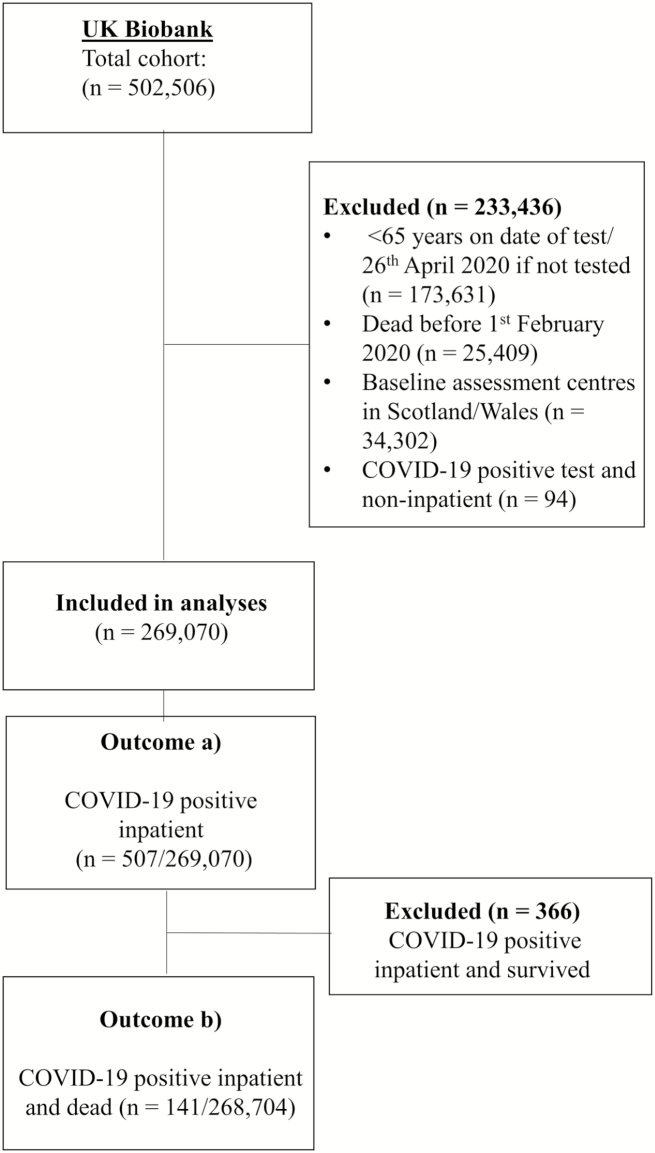
Flowchart of UK Biobank participants selected for analyses.

Data were from UKB participants with linked data on COVID-19 infection (by polymerase chain reaction) from tests performed between March 16 and April 26, 2020, in England only (14) and linked data on death records to April 26, 2020. We excluded participants who tested positive for COVID-19 but were not recorded as being hospital inpatients on the test sample submitted to the laboratory. We restricted analyses to participants aged 65 years and older when tested (or age on April 26, 2020 if not tested), to minimize misclassification of disease severity from health care workers. We also excluded UKB participants reported to have died before the pandemic (set as February 1, 2020). No COVID-19 test data were available for UKB assessment centers in Scotland and Wales, so data from these centers were not included. Our outcomes of interest were (a) COVID-19 test positive compared to the rest of the sample (ie, test negative and untested samples) and (b) COVID-19 test positive and died versus the rest of the sample, but with the additional exclusion of participants testing positive but who were still alive (see Figure 1 for details of UKB participants included in the analyses).

### Disease Ascertainment

Preexisting diagnoses were available from baseline questionnaires (2006–2010) eliciting participant reports of doctor-diagnosed disease. New disease diagnoses since baseline were from linked electronic medical records to hospital inpatient routine data (to March 2017), coded according to the International Classification of Diseases 10th revision (ICD-10). Diagnoses included were coronary heart disease (CHD), atrial fibrillation, stroke, hypertension, diabetes (type 2), chronic kidney disease (CKD, stages 3–5), depression, dementia, asthma, chronic obstructive pulmonary disease (COPD), osteoporosis, and osteoarthritis. We also identified previous diagnoses of delirium, pneumonia, and falls or fragility fractures (see definitions in Supplementary Table 1). We combined each diagnosis reported at baseline or from linked hospital data to generate preexisting diagnosis status for each participant.

### Statistical Analysis

We estimated associations of demographic and diagnoses with inpatient COVID-19 test positivity (alone or with mortality) using logistic regression models, with 95% CIs. Logistic models were adjusted for age group (in 5-year bands), sex, ethnicity, education, and assessment center at baseline (to account for geographic differences in the prevalence of COVID-19 infection). We performed subgroup analyses stratified by sex and tested for interaction terms by sex. We also performed sensitivity analyses, comparing inpatient COVID-19 test positives with those testing negative. A *p* value smaller than 5% was considered statistically significant. All the statistical analyses were performed in Stata version 15.1.

## Results

There were 269 070 older adults (aged 65–86 years, mean 73.1 years) eligible for the analysis, of whom 507 (0.2%) were laboratory test-positive COVID-19 hospital inpatients, including 141 (27.8%) certified to have died. Rates of COVID-19 hospitalization varied widely across UKB baseline assessment centers (0.34%–0.07%; Supplementary Table 2). The mean age of COVID-19 test-positive inpatients (Table 1) was 74.3 years (*SD* 4.5), versus 73.1 years (*SD* 4.4) for other study participants. Both inpatients and others were predominantly of “white” ethnicity, but people self-reporting “black” ethnicity made up 3.6% of inpatients but only 1% of participants. The most common preexisting diagnoses in inpatients were hypertension (59.6%), a history of falls or fragility fractures (29.4%), CHD (21.5%), diabetes (type 2, 19.9%), and asthma (17.6%). Dementia was present in 2.8% of inpatients (0.3% of other participants). COVID-19 inpatients had a mean of 2.3 preexisting diagnoses (of a possible 15 examined) compared to other participants with mean 1.4 diagnoses.

**Table 1.** Descriptive Characteristics of UK Biobank Cohort by Inpatient COVID-19 Test Positivity

	Inpatient COVID-19 Positive Test			Rest of the Cohort		
	Men	Women	Total	Men	Women	Total
Total number	311	196	507	121 096	147 467	268 563
Dead	95 (30.6)	46 (23.5)	141 (27.8)	471 (0.4)	344 (0.2)	815 (0.3)
Age (years), mean (*SD*)	74.7 (4.4)	73.6 (4.6)	74.3 (4.5)	73.3 (4.4)	73.0 (4.4)	73.1 (4.4)
65–69	46 (14.8)	51 (26.0)	97 (19.1)	31 522 (26.0)	41 174 (27.9)	72 696 (27.1)
70–74	102 (32.8)	64 (32.7)	166 (32.7)	44 117 (36.4)	55 532 (37.7)	99 649 (37.1)
75–79	132 (42.4)	65 (33.2)	197 (38.9)	37 642 (31.3)	42 138 (28.6)	79 780 (29.7)
80+	31 (10.0)	16 (8.2)	47 (9.3)	7815 (6.5)	8623 (5.9)	16 438 (6.1)
Ethnicity						
White	282 (92.2)	171 (87.7)	453 (90.4)	115 714 (96.2)	141 152 (96.1)	256 866 (96.2)
Black	10 (3.3)	8 (4.1)	18 (3.6)	1108 (0.9)	1618 (1.1)	2726 (1.0)
South Asian	6 (2.0)	11 (5.6)	17 (3.4)	2204 (1.8)	1996 (1.4)	4200 (1.6)
Other (including mixed and Chinese)	8 (2.6)	5 (2.6)	13 (2.6)	1278 (1.1)	2057 (1.4)	3335 (1.3)
Education						
None	119 (40.2)	75 (39.3)	194 (39.8)	25 008 (21.1)	32 786 (22.7)	57 794 (22.0)
School/College	71 (24.0)	52 (27.2)	123 (25.3)	37 172 (31.4)	48 430 (33.6)	85 602 (32.6)
Professional qualification	45 (15.2)	26 (13.6)	71 (14.6)	17 763 (15.0)	24 001 (16.6)	41 764 (15.9)
Degree	61 (20.6)	38 (19.9)	99 (20.3)	38 514 (32.5)	39 044 (27.1)	77 558 (29.5)
Prevalent disease*						
CHD	80 (25.7)	29 (14.8)	109 (21.5)	19 986 (16.5)	10 949 (7.4)	30 935 (11.5)
Atrial fibrillation	53 (17.0)	15 (7.7)	68 (13.4)	9164 (7.6)	5097 (3.5)	14 261 (5.3)
Stroke	15 (4.8)	8 (4.1)	23 (4.5)	3176 (2.6)	2227 (1.5)	5403 (2.0)
Hypertension	194 (62.4)	108 (55.1)	302 (59.6)	55 772 (46.1)	53 981 (36.6)	109 753 (40.9)
Diabetes (type 2)	71 (22.8)	30 (15.3)	101 (19.9)	11 004 (9.1)	7518 (5.1)	18 522 (6.9)
Chronic kidney disease	10 (3.2)	13 (6.6)	23 (4.5)	1814 (1.5)	2061 (1.4)	3875 (1.4)
Depression	38 (12.2)	35 (17.9)	73 (14.4)	6724 (5.6)	12 749 (8.7)	19 473 (7.3)
Dementia	7 (2.3)	7 (3.6)	14 (2.8)	412 (0.3)	462 (0.3)	874 (0.3)
Asthma	39 (12.5)	51 (26.0)	90 (17.6)	14 100 (11.6)	20 512 (13.9)	34 612 (12.9)
COPD	36 (11.6)	26 (13.3)	62 (12.2)	5790 (4.8)	5656 (3.8)	11 446 (4.3)
Osteoporosis	9 (2.9)	15 (7.7)	24 (4.7)	1160 (1.0)	6309 (4.3)	7469 (2.8)
Osteoarthritis	45 (14.5)	41 (20.9)	86 (17.0)	12 987 (10.7)	23 553 (16.0)	36 540 (13.6)
Previous disease/condition*						
Delirium	4 (1.3)	1 (0.5)	5 (1.0)	320 (0.3)	284 (0.2)	604 (0.2)
Pneumonia	40 (12.9)	18 (9.2)	58 (11.4)	5146 (4.3)	5051 (3.4)	10 197 (3.8)
Falls/Fragility fractures	82 (26.4)	67 (34.2)	149 (29.4)	22 531 (18.6)	44 302 (30.0)	66 833 (24.9)
Number of above diseases (max 15), mean (*SD*)	2.3 (1.8)	2.4 (1.8)	2.3 (1.8)	1.4 (1.4)	1.4 (1.3)	1.4 (1.4)
Number of hospital admissions, mean (*SD*)	5.9 (10.4)	5.1 (7.2)	5.6 (9.3)	3.0 (9.3)	2.7 (5.8)	2.8 (7.6)

*Notes:* CHD = coronary heart disease; COPD = chronic obstructive pulmonary disease. The numbers presented are *n* (%) unless otherwise specified. The rest of the cohort includes test negative and untested UK Biobank participants. *Diagnoses from baseline self-report and hospital inpatient admissions.

In logistic modeling of demographic variables (Table 2, “Demographics”), rates of COVID-19 hospitalization were higher in those aged older than 80 years (odds ratio [OR] = 2.02, 95% CI: 1.41–2.89, *p* = 1.40E-04) compared to age group 65–69, with no statistically significant risk increase in 70–74 years old and intermediate risks in 75–79 years old. Males were substantially more likely to be COVID-19 test-positive patients (OR = 1.91, 95% CI: 1.59–2.29, *p* = 5.10E-12) and people of black ethnicity were at higher risk (OR = 3.17, 95% CI: 1.92–5.25, *p* = 7.20E-06) compared to white, with South Asians and other ethnicities having intermediate risks. Compared to those with degree-level education, having no education qualifications (OR = 2.52, 95% CI: 1.96–3.24, *p* = 5.80E-13) was associated with raised risks of COVID-19.

**Table 2.** Risk of Hospitalized COVID-19 and Mortality by Demographic Variables and Preexisting Diagnoses

	COVID-19 Positive Inpatient				COVID-19 Positive Inpatient and Dead*			
	Demographics		Full Model		Demographics		Full Model	
	OR (95% CI)	*p* Value	OR (95% CI)	*p* Value	OR (95% CI)	*p* Value	OR (95% CI)	*p* Value
Age (years)								
65–69	1.00		1.00		1.00		1.00	
70–74	1.17 (0.91–1.52)	2.30E-01	1.12 (0.86–1.45)	4.00E-01	1.24 (0.71–2.18)	4.50E-01	1.15 (0.65–2.02)	6.30E-01
75–79	1.60 (1.24–2.06)	2.70E-04	1.40 (1.08–1.81)	1.00E-02	2.61 (1.55–4.39)	3.00E-04	2.16 (1.27–3.65)	4.30E-03
80+	2.02 (1.41–2.89)	1.40E-04	1.60 (1.11–2.32)	1.20E-02	3.66 (1.87–7.18)	1.50E-04	2.65 (1.33–5.29)	5.60E-03
Sex								
Female	1.00		1.00		1.00		1.00	
Male	1.91 (1.59–2.29)	5.10E-12	1.79 (1.48–2.17)	2.50E-09	2.36 (1.65–3.39)	2.90E-06	2.12 (1.45–3.09)	1.00E-04
Ethnicity								
White	1.00		1.00		1.00		1.00	
Black	3.17 (1.92–5.25)	7.20E-06	2.85 (1.71–4.74)	5.60E-05	3.32 (1.42–7.77)	5.70E-03	2.60 (1.09–6.19)	3.00E-02
South Asian	2.01 (1.20–3.36)	8.30E-03	1.69 (1.00–2.85)	5.00E-02	1.63 (0.59–4.51)	3.50E-01	1.20 (0.42–3.36)	7.40E-01
Other (including mixed and Chinese)	2.22 (1.24–3.98)	7.50E-03	2.00 (1.11–3.61)	2.00E-02	No observations		No observations	
Education								
Degree	1.00		1.00		1.00		1.00	
Professional qualification	1.40 (1.03–1.91)	3.40E-02	1.31 (0.96–1.79)	8.80E-02	1.66 (0.93–2.97)	8.50E-02	1.51 (0.85–2.70)	1.60E-01
School/College	1.20 (0.92–1.57)	1.90E-01	1.11 (0.85–1.45)	4.50E-01	1.23 (0.73–2.08)	4.30E-01	1.09 (0.64–1.84)	7.60E-01
None	2.52 (1.96–3.24)	5.80E-13	2.06 (1.60–2.66)	2.70E-08	2.43 (1.49–3.97)	3.80E-04	1.82 (1.10–2.99)	1.90E-02
Prevalent disease^†^								
CHD			0.95 (0.74–1.21)	6.60E-01			0.86 (0.55–1.36)	5.30E-01
Atrial fibrillation			1.64 (1.24–2.17)	5.00E-04			1.63 (0.98–2.71)	5.80E-02
Stroke			1.16 (0.75–1.81)	5.00E-01			0.93 (0.40–2.17)	8.70E-01
Hypertension			1.38 (1.13–1.68)	1.70E-03			1.42 (0.96–2.11)	7.90E-02
Diabetes (type 2)			1.73 (1.36–2.22)	1.20E-05			3.11 (2.06–4.71)	7.60E-08
Chronic kidney disease			1.49 (0.96–2.31)	7.30E-02			0.88 (0.35–2.24)	8.00E-01
Depression			1.79 (1.37–2.33)	1.70E-05			1.78 (1.07–2.96)	2.70E-02
Dementia			3.50 (1.93–6.34)	3.60E-05			7.30 (3.28–16.21)	1.10E-06
Asthma			1.12 (0.87–1.44)	3.70E-01			0.59 (0.33–1.04)	6.70E-02
COPD			1.58 (1.17–2.15)	2.90E-03			1.91 (1.10–3.32)	2.20E-02
Osteoporosis			1.40 (0.91–2.14)	1.20E-01			1.36 (0.58–3.17)	4.80E-01
Osteoarthritis			0.98 (0.77–1.26)	8.90E-01			1.08 (0.69–1.70)	7.40E-01
Previous disease/ condition^†^								
Delirium			1.14 (0.45–2.90)	7.80E-01			1.02 (0.23–4.58)	9.70E-01
Pneumonia			1.96 (1.45–2.64)	1.10E-05			1.88 (1.07–3.30)	2.80E-02
Falls/Fragility fractures			1.10 (0.89–1.34)	3.80E-01			1.20 (0.82–1.76)	3.40E-01

*Notes:* CHD = coronary heart disease; CI = confidence interval; COPD = chronic obstructive pulmonary disease; OR = odds ratio. Demographics model (adjusted for age group, sex, ethnicity, education, and baseline assessment center). Full model (adjusted for age group, sex, ethnicity, education, baseline assessment center, and all the above diseases/conditions). *Comparison group excluded participants testing positive and surviving. ^†^Diagnoses from baseline self-report and hospital inpatient admissions.

All studied diagnoses individually (adjusted for demographics only) were associated with a COVID-19 positive test, with the exception of osteoarthritis (Supplementary Table 3). As different chronic diseases often coexist in older adults, we estimated risks for each diagnosis accounting for other diagnoses present. In models adjusted for demographics and the other studied preexisting diagnoses (Table 2, Full model), dementia was associated with the largest increase in risk of COVID-19 hospitalization (OR = 3.50, 95% CI: 1.93–6.34, *p* = 4.3.60E-05), followed by pneumonia (OR = 1.96, 95% CI: 1.45–2.64, *p* = 1.10E-05), depression (OR = 1.79, 95% CI: 1.37–2.33, *p* = 1.70E-05), diabetes (OR = 1.73, 95% CI: 1.36–2.22, *p* = 1.20E-05), atrial fibrillation (OR = 1.64, 95% CI: 1.24–2.17, *p* = 5.00E-04), and COPD (OR = 1.58, 95% CI: 1.17–2.15, *p* = 2.90E-03), with a modest risk increase with hypertension (OR = 1.38, 95% CI: 1.13–1.68, *p* = 1.70E-03). Coronary heart disease prevalence, previously noted as common in COVID-19 inpatients, did not differ between inpatients and other participants (OR = 0.95, 95% CI: 0.74–1.21, *p* = 6.60E-01) after adjustment for other diagnoses.

In logistic models for the risk of COVID-19 hospitalization and death, preexisting dementia was associated with the largest risk increase (OR = 7.30, 95% CI 3.28–16.21, *p* = 1.10E-06), followed by diabetes (OR = 3.11, 95% CI: 2.06–4.71, *p* = 7.60E-08), COPD (OR = 1.91, 95% CI: 1.10–3.32, *p* = 2.20E-02), pneumonia (OR = 1.88, 95% CI: 1.07–3.30, *p* = 2.80E-02), and depression (OR = 1.78, 95% CI: 1.07–2.96, *p* = 2.70E-02). Although prevalent atrial fibrillation and hypertension were significantly associated with COVID-19 hospitalization, these diagnoses did not reach a significance for being a COVID-19 inpatient and dying (*p* > .05; Table 2).

In sex-specific analyses (Supplementary Table 4) of COVID-19 hospitalization, significant interactions by sex were only present for CKD (*p* = 4.20E-02) and asthma (*p* = 1.10E-02), which were both risk factors in women but not in men. For inpatient COVID-19 positivity and death, a significant interaction by sex was only present for CKD (*p* = 3.80E-02), which was a significant risk factor in women only.

## Discussion

We aimed to identify risk factors in older UKB participants associated with hospitalization with COVID-19, alone or with mortality, during the peak of the initial epidemic in England. Preexisting diagnoses of dementia, type 2 diabetes, COPD, pneumonia, and depression, as well as atrial fibrillation and hypertension emerged as independent risk factors for COVID-19 hospitalization, the first 5 remaining statistically significant for related mortality. In addition, preexisting asthma and CKD emerged as risk factors in women for being hospitalized with COVID-19. Interestingly, CHD was common in COVID-19 inpatients and associated with COVID-19 in models adjusted for demographics only, but this association was no longer present after adjustment for comorbidities. In keeping with other studies, we also found an increased risk of COVID-19 in males, but we have shown that this risk is virtually unchanged after adjusting for comorbidities. In addition, we confirmed previous reports that people of black ethnicity and those with no educational qualifications had a higher risk. Overall, these results suggest that there are specific risk comorbidities in older groups and that severe COVID-19 susceptibility is not merely the result of advancing age.

Hypertension is well recognized as the most common chronic diagnosis, prevalent in more than 70% of persons at age older than 80 (15). While highly prevalent in the UKB cohort, we found that it was only modestly more common in COVID-19 cases than in other UKB participants and not significantly associated with COVID-19 positivity and death. Interestingly, in females, we found that having a diagnosis of CKD (Grade 3–5) was associated with being COVID-19 positive (alone and with mortality), mirroring previous analyses by Masoli et al. (16), who found that CKD grade is more predictive of mortality than blood pressure in adults aged older than 70. Chronic kidney disease has also been reported to be associated with increased hospitalization with infection, particularly pneumonia, and increased 30-day mortality (17,18).

Another novel finding is the association between atrial fibrillation and hospitalized COVID-19 positivity in the studied older adults and an association with COVID-19 positive and dead in men specifically. During atrial fibrillation, the loss of atrioventricular synchrony with decreased diastolic filling time is likely to lead to a decrease in cardiac output. Consequently, this low cardiac output may aggravate tissue hypoxia in COVID-19 patients. Also, agents used in the control of atrial fibrillation, particularly sotalol, propafenone, and nonselective β-blockers, may cause bronchospasm (19). Pulmonary symptoms in COPD may become worse with atrial fibrillation development, due to excessive irregular heart rate, as well as the reduced diastolic filling of the ventricles (20). These factors may contribute to higher severe COVID-19 risk in participants with atrial fibrillation.

To date, there has been limited data on preexisting diagnoses of dementia and COVID-19 hospitalization, despite dementia affecting more than 50 million people worldwide (21). This may be due to the young median ages of many published COVID-19 case series, with a limited characterization of older persons. Our analysis confirms dementia to be the largest effect risk diagnosis in adults aged older than 65 for risk of hospitalized COVID-19 test positivity and death, in this cohort of community volunteers. A recent report of observational data from the International Severe Acute Respiratory and Emerging Infections Consortium found a high prevalence of dementia in older adults admitted to hospital with COVID-19 (3). Future work will need to establish whether this a direct effect of dementia pathologies or an indirect effect of high rates of infection in nursing homes. Recent studies have reported that the APOE e4 genotype (a risk factor for Alzheimer’s disease) predicts severe COVID-19 (22) and death (23), independent of preexisting dementia, but further work is needed to understand the biological mechanisms involved. Further work is also needed to establish the extent to which the observed association between depression and COVID-19 hospitalization is due to the depression itself or secondary to other diagnosed and undiagnosed physical pathologies.

In this analysis, we compared the characteristics of older COVID-19 inpatients to the rest of the study population, aiming to identify predictive factors for a severe infection that might be used to identify older people at higher risk in the community. An alternative analysis approach could compare COVID-19-positive inpatients (*n* = 507) to inpatients whose COVID-19 tests were negative (*n* = 596) during the study period (Supplementary Table 5). However, it should be noted that during the period studied at the epidemic peak, COVID-19 testing was largely restricted to hospital inpatients with symptoms of the infection (14), and the polymerase chain reaction test used had a significant false-negative rate of up to 29% on initial testing (24). Differences between test positives and negatives may therefore reflect other reasons for hospital admission with symptoms resembling COVID-19, plus false negatives. In the test-positive versus test-negative analysis (Supplementary Table 5), prevalent diabetes was associated with an increased risk, and a previous delirium episode was associated with a decreased risk of a positive COVID-19 test, and dementia and diabetes were associated with an increased risk of a positive COVID-19 test and death. As the statistical power to detect test-positive versus negative differences is far lower than in the comparison with the rest of the UKB sample, caution is needed in interpreting nonsignificant associations with the high-risk diseases identified in the main analysis.

UK Biobank participants were somewhat healthier than the general population (25) at baseline in 2006–2010, but the sample nevertheless includes large numbers of socioeconomically less privileged participants: for example, 36.1% of cases and 22.3% of controls had no educational qualifications (Table 1). Other limitations include the lack of details of degrees of clinical severity of COVID-19, beyond the ascertained measures of hospitalization and mortality. We do not currently have access to measures of COVID-19 exposure in the UKB sample or details of COVID-19 illnesses that did not result in hospital admission. During the peak of the epidemic, some patients with, for example, angina or even myocardial infarction may have been deterred from seeking medical help (26), which may possibly have influenced our finding of no association between CHD and COVID-19 hospitalization in fully adjusted models. However, CHD was more common in COVID-19 hospitalization on simple adjustment for demographics, with this association disappearing only after adjustment for other diagnoses. It may therefore be that common related comorbidities, for example, atrial fibrillation, hypertension, and CKD, are actually more predictive of COVID-19 hospitalization than preexisting CHD itself in older people. There may have been under ascertainment of inpatient COVID-19-related deaths in the data available for April 2020 (27), but as the analyses for risk of COVID-19 positivity and death excluded participants who tested positive and survived, the impact on mortality associations is likely to be small. UK Biobank does not have data on care home residence, but the sample is relatively young (93.9% <80 years), and at the peak of the epidemic hospital admission from care homes was restricted (14).

Only a small proportion of the English population had been exposed to the virus during the study period, but the group studied here was exposed and developed severe enough COVID-19 to be tested during hospitalization, with some of those dying. Our case group is therefore relevant for assessing risk factors for severe COVID-19 inpatients and death in this older population, but may not apply to asymptomatic or milder nonhospitalized cases. Our diagnostic data are derived from participant’s baseline interviews plus hospital discharge data until March 2017, so under-ascertainment of disease is likely, especially for recently diagnosed conditions, but the similarity to previous reports of the common conditions seen in COVID-19 patients suggests that our data are valid.

Our results should have implications for preventive interventions, encouraging a more targeted approach prioritizing those older adults with specific risk factors, rather than adopting policies that use chronological older age as a blanket indicator of risk. Our cohort evidence of specific risk factors may also help with avoiding potentially “ageist” approaches to setting clinical priorities in over-stretched health systems (28). Our findings of risks associated with less prominent conditions such as atrial fibrillation and depression, plus asthma and CKD in women only, could help focus clinical research. In addition, the prominence of depression as one of the major risk factors highlights the role of mental health (29) as critical to managing the pandemic, including in older people.

## Conclusions

In older adults, several specific preexisting comorbidities are disproportionally common in hospitalized COVID-19 male and female patients, alone or with mortality, notably including dementia, depression, atrial fibrillation, and CKD. Clinical and public health research is needed to establish the mechanisms involved and whether stratified interventions are needed for older patients with specific comorbidities. Our results do not support the simple age targeting of interventions to prevent severe COVID-19 infection.

## Funding

UK Medical Research Council award MR/S009892/1 (PI, Melzer) supports J.L.A. D.M. and L.C.P. are supported by the University of Exeter Medical School and, in part, by the University of Connecticut School of Medicine. J.A.H.M. is supported by the NIHR Doctoral Research Fellowship (DRF-2014-07-177). Input from C.L.K. was supported by the University of Connecticut. The views expressed in this publication are those of the author(s) and not necessarily those of the NHS, the National Institute for Health Research or the Department of Health.

## Supplementary Material

glaa183_suppl_Supplementary_Tables
