# Supplementary material for: Preexisting Comorbidities Predicting COVID-19 and Mortality in the UK Biobank Community Cohort
Source: J Gerontol A Biol Sci Med Sci. 2020 Jul 20;75(11):2224–30. doi: 10.1093/gerona/glaa183 (PMC7454409; doi:10.1093/gerona/glaa183)
Supplement: glaa183_suppl_Supplementary_Tables [file glaa183_suppl_supplementary_tables.docx]

## **preexisting comorbidities predicting COVID-19 AND MORTALITY in the UK Biobank community cohort**

**Atkins et al, JG:MS 2020**

**Supplementary Tables**

## **Supplementary Table 1.** Details of included diseases; International Classification of Diseases 10th revision (ICD-10) codes included from inpatient hospital records

|  | **ICD-10 Codes** |
| --- | --- |
| **Prevalent disease** |  |
| Coronary heart disease | I20–I25 |
| Atrial fibrillation | I48 |
| Stroke | G45-G46; I61; I63 |
| Hypertension | I10-I15 |
| Diabetes (Type 2) | E11 |
| Chronic kidney disease | N18; N183; N184; N185; Y841 |
| Depression | F32; F33; F34.1 |
| Dementia | F00; F01; F02; F03; G30 |
| Asthma | J45-J46 |
| Chronic obstructive pulmonary disease | J42-J44 |
| Osteoporosis | M80; M81; M811; M812; M813; M814; M815; M816; M818; M819 |
| Osteoarthritis | M15.0; M15.1; M15.2; M15.9; M16.0; M16.1; M17.0; M17.1; M18.0; M18.1; M19.0 |
|  |  |
| **Previous disease/condition** |  |
| Delirium | F05 |
| Pneumonia | J13; J14; J15; J16; J17; J18 |
| Falls/Fragility fractures | W00; W01; W04; W05; W06; W07; W08; W10; W17; W18; W19; R296 |
|  | S220; S32; S325; S328; S422-S424; S524; S525; S720-S722; S582; S5823; T08 |

## **Supplementary Table 2**. Number (%) of positive COVID-19 inpatients by baseline UK Biobank assessment center

|  | **COVID-19 positive inpatient** | | | | | |
| --- | --- | --- | --- | --- | --- | --- |
|  | **Men** | | **Women** | | **Total** | |
| **Assessment centre** | **n** | **%** | **n** | **%** | **n** | **%** |
|  |  |  |  |  |  |  |
| Barts | 16 | 0.56 | 5 | 0.14 | 21 | 0.32 |
| Birmingham | 25 | 0.37 | 11 | 0.14 | 36 | 0.25 |
| Bristol | 15 | 0.14 | 8 | 0.06 | 23 | 0.09 |
| Bury | 21 | 0.25 | 7 | 0.07 | 28 | 0.16 |
| Croydon | 20 | 0.29 | 14 | 0.16 | 34 | 0.21 |
| Hounslow | 25 | 0.34 | 24 | 0.26 | 49 | 0.30 |
| Leeds | 23 | 0.19 | 18 | 0.12 | 41 | 0.15 |
| Liverpool | 28 | 0.31 | 23 | 0.21 | 51 | 0.25 |
| Manchester | 18 | 0.49 | 7 | 0.16 | 25 | 0.31 |
| Middlesbrough | 15 | 0.25 | 14 | 0.20 | 29 | 0.22 |
| Newcastle | 29 | 0.28 | 27 | 0.21 | 56 | 0.24 |
| Nottingham | 18 | 0.19 | 13 | 0.11 | 31 | 0.15 |
| Oxford | 4 | 0.11 | 4 | 0.08 | 8 | 0.09 |
| Reading | 5 | 0.06 | 7 | 0.07 | 12 | 0.07 |
| Sheffield | 24 | 0.28 | 8 | 0.08 | 32 | 0.17 |
| Stockport | 7 | 0.68 | 1 | 0.08 | 8 | 0.34 |
| Stoke | 18 | 0.29 | 5 | 0.08 | 23 | 0.19 |
|  |  |  |  |  |  |  |
| Total | 311 | 0.26 | 196 | 0.13 | 507 | 0.19 |

National Health Service COVID-19 laboratory test results for inpatients in England for UK Biobank participants for the period March 16 to April 26 2020. Proportion presented above compares positive tests results to the rest of UK Biobank cohort (aged 65 years or above, alive on 1^st^ February 2020 and attending baseline assessment centers in England).

## **Supplementary Table 3**. Risk of hospitalized COVID-19 and mortality by pre-existing diagnoses, accounting for demographics

|  | **COVID-19 Positive inpatient** | |  | **COVID-19 Positive inpatient and Dead**** | |
| --- | --- | --- | --- | --- | --- |
|  | **Demographics** | |  | **Demographics** | |
|  | **OR (95% CI)** | **p-value** |  | **OR (95% CI)** | **p-value** |
| Prevalent disease* |  |  |  |  |  |
| CHD | 1.50 (1.20 to 1.88) | 3.80E-04 |  | 1.52 (1.00 to 2.31) | 4.80E-02 |
| Atrial fibrillation | 2.28 (1.75 to 2.97) | 8.80E-10 |  | 2.39 (1.48 to 3.84) | 3.30E-04 |
| Stroke | 1.81 (1.17 to 2.78) | 7.10E-03 |  | 1.63 (0.71 to 3.71) | 2.50E-01 |
| Hypertension | 1.73 (1.43 to 2.08) | 8.50E-09 |  | 1.97 (1.37 to 2.83) | 2.60E-04 |
| Diabetes (type 2) | 2.40 (1.9 to 3.02) | 1.40E-13 |  | 4.15 (2.83 to 6.1) | 3.70E-13 |
| Chronic kidney disease | 2.64 (1.73 to 4.04) | 7.10E-06 |  | 1.97 (0.80 to 4.84) | 1.40E-01 |
| Depression | 2.33 (1.8 to 3.01) | 9.30E-11 |  | 2.52 (1.54 to 4.11) | 2.30E-04 |
| Dementia | 6.28 (3.58 to 11.03) | 1.50E-10 |  | 13.18 (6.33 to 27.42) | 5.30E-12 |
| Asthma | 1.50 (1.18 to 1.89) | 7.10E-04 |  | 0.88 (0.51 to 1.50) | 6.30E-01 |
| COPD | 2.45 (1.86 to 3.23) | 1.90E-10 |  | 2.59 (1.56 to 4.30) | 2.20E-04 |
| Osteoporosis | 1.90 (1.25 to 2.89) | 2.60E-03 |  | 1.84 (0.80 to 4.23) | 1.50E-01 |
| Osteoarthritis | 1.17 (0.92 to 1.49) | 2.10E-01 |  | 1.29 (0.82 to 2.02) | 2.70E-01 |
|  |  |  |  |  |  |
| Previous disease/condition* |  |  |  |  |  |
| Delirium | 3.41 (1.4 to 8.29) | 6.90E-03 |  | 4.37 (1.07 to 17.83) | 4.00E-02 |
| Pneumonia | 2.90 (2.19 to 3.85) | 1.10E-13 |  | 2.95 (1.74 to 4.99) | 5.60E-05 |
| Falls/Fragility fractures | 1.31 (1.07 to 1.59) | 7.70E-03 |  | 1.48 (1.02 to 2.14) | 3.80E-02 |

Demographics model (adjusted for age group, sex, ethnicity, education, and baseline assessment centre)

*Diagnoses from baseline self-report and hospital inpatient admissions

**Comparison group excluded participants testing positive and surviving.

## **Supplementary Table 4**. Risk of hospitalized COVID-19 and mortality by pre-existing diagnoses, stratified by sex

|  | | **COVID Positive** | | | | |  |  | **COVID Positive and Dead**** | | | | |  |
| --- | --- | --- | --- | --- | --- | --- | --- | --- | --- | --- | --- | --- | --- | --- |
|  | | **WOMEN** | |  | **MEN** | | **p-value for interaction** |  | **WOMEN** | |  | **MEN** | | **p-value for interaction** |
|  | | **Full adjustment** | |  | **Full adjustment** | |  |  | **Full adjustment** | |  | **Full adjustment** | |  |
|  | **OR (95% CI)** | | **p-value** |  | **OR (95% CI)** | **p-value** |  |  | **OR (95% CI)** | **p-value** |  | **OR (95% CI)** | **p-value** |  |
| **Prevalent disease*** | |  |  |  |  |  |  |  |  |  |  |  |  |  |
| CHD | | 0.90 (0.57 to 1.42) | 6.50E-01 |  | 0.97 (0.73 to 1.29) | 8.40E-01 | 9.50E-01 |  | 1.5 (0.65 to 3.46) | 3.40E-01 |  | 0.70 (0.41 to 1.2) | 2.00E-01 | 3.90E-01 |
| Atrial fibrillation | | 1.31 (0.73 to 2.34) | 3.60E-01 |  | 1.78 (1.29 to 2.45) | 4.50E-04 | 3.70E-01 |  | 0.28 (0.04 to 2.15) | 2.20E-01 |  | 2.18 (1.27 to 3.75) | 5.00E-03 | 7.70E-02 |
| Stroke | | 1.33 (0.61 to 2.90) | 4.80E-01 |  | 1.10 (0.65 to 1.89) | 7.20E-01 | 7.10E-01 |  | 1.81 (0.42 to 7.83) | 4.30E-01 |  | 0.74 (0.26 to 2.09) | 5.70E-01 | 5.90E-01 |
| Hypertension | | 1.48 (1.09 to 2.03) | 1.30E-02 |  | 1.30 (1.00 to 1.68) | 4.60E-02 | 6.00E-01 |  | 1.48 (0.77 to 2.83) | 2.40E-01 |  | 1.36 (0.83 to 2.23) | 2.20E-01 | 9.70E-01 |
| Diabetes (type 2) | | 1.55 (1.00 to 2.40) | 5.10E-02 |  | 1.84 (1.37 to 2.48) | 5.80E-05 | 9.80E-01 |  | 1.86 (0.8 to 4.34) | 1.50E-01 |  | 3.69 (2.28 to 5.99) | 1.20E-07 | 3.30E-01 |
| Chronic kidney disease | | 2.51 (1.38 to 4.56) | 2.60E-03 |  | 0.96 (0.50 to 1.85) | 9.10E-01 | 4.20E-02 |  | 3.41 (1.15 to 10.09) | 2.70E-02 |  | 0.22 (0.03 to 1.63) | 1.40E-01 | 3.80E-02 |
| Depression | | 1.75 (1.19 to 2.58) | 4.80E-03 |  | 1.81 (1.26 to 2.61) | 1.30E-03 | 8.70E-01 |  | 2.24 (1.04 to 4.79) | 3.80E-02 |  | 1.50 (0.75 to 3.01) | 2.50E-01 | 5.60E-01 |
| Dementia | | 6.88 (3.06 to 15.48) | 3.20E-06 |  | 2.28 (0.96 to 5.40) | 6.10E-02 | 1.70E-01 |  | 8.29 (1.88 to 36.63) | 5.30E-03 |  | 8.09 (3.14 to 20.84) | 1.50E-05 | 6.50E-01 |
| Asthma | | 1.52 (1.07 to 2.16) | 2.00E-02 |  | 0.85 (0.59 to 1.22) | 3.70E-01 | 1.10E-02 |  | 0.67 (0.27 to 1.66) | 3.90E-01 |  | 0.53 (0.25 to 1.10) | 8.90E-02 | 9.30E-01 |
| COPD | | 1.96 (1.22 to 3.15) | 5.20E-03 |  | 1.43 (0.97 to 2.12) | 7.30E-02 | 1.90E-01 |  | 1.04 (0.29 to 3.64) | 9.60E-01 |  | 2.24 (1.20 to 4.20) | 1.20E-02 | 8.10E-04 |
| Osteoporosis | | 1.22 (0.70 to 2.11) | 4.80E-01 |  | 1.88 (0.95 to 3.71) | 7.00E-02 | 4.20E-01 |  | 1.18 (0.36 to 3.89) | 7.90E-01 |  | 1.96 (0.60 to 6.44) | 2.70E-01 | 4.00E-01 |
| Osteoarthritis | | 0.98 (0.68 to 1.41) | 9.00E-01 |  | 0.97 (0.69 to 1.35) | 8.40E-01 | 9.80E-01 |  | 1.37 (0.69 to 2.72) | 3.70E-01 |  | 0.90 (0.48 to 1.67) | 7.40E-01 | 4.10E-01 |
| **Previous disease/condition*** | |  |  |  |  |  |  |  |  |  |  |  |  |  |
| Delirium | | 0.40 (0.05 to 3.25) | 3.90E-01 |  | 1.63 (0.58 to 4.60) | 3.60E-01 | 3.80E-01 |  | No observation |  |  | 1.50 (0.33 to 6.94) | 6.00E-01 | No observation |
| Pneumonia | | 1.76 (1.05 to 2.96) | 3.10E-02 |  | 2.07 (1.44 to 2.99) | 9.20E-05 | 7.70E-01 |  | 2.06 (0.70 to 6.05) | 1.90E-01 |  | 1.84 (0.95 to 3.53) | 6.90E-02 | 7.60E-01 |
| Falls/Fragility fractures | | 0.96 (0.70 to 1.31) | 7.90E-01 |  | 1.22 (0.93 to 1.59) | 1.40E-01 | 3.20E-01 |  | 0.92 (0.48 to 1.73) | 7.90E-01 |  | 1.40 (0.88 to 2.23) | 1.60E-01 | 2.60E-01 |

Full model (adjusted for age group, ethnicity, education, baseline assessment centre and all the above diseases/conditions).

*Diagnoses from baseline self-report and hospital inpatient admissions

**Comparison group excluded participants testing positive and surviving.

## **Supplementary Table 5**. Risk of hospitalized COVID-19 and mortality by pre-existing diagnoses, comparing individuals who tested positive to those who tested negative

|  | **COVID19-Positive inpatient** | |  | **COVID-19 Positive inpatient and Dead**** | |
| --- | --- | --- | --- | --- | --- |
|  | **Full adjustment** | |  | **Full adjustment** | |
|  | **OR (95% CI)** | **p-value** |  | **OR (95% CI)** | **p-value** |
| **Prevalent disease*** |  |  |  |  |  |
| CHD | 0.75 (0.52 to 1.06) | 1.00E-01 |  | 0.65 (0.37 to 1.14) | 1.40E-01 |
| Atrial fibrillation | 1.23 (0.82 to 1.86) | 3.20E-01 |  | 1.03 (0.54 to 1.95) | 9.30E-01 |
| Stroke | 0.85 (0.45 to 1.58) | 6.00E-01 |  | 0.64 (0.23 to 1.78) | 3.90E-01 |
| Hypertension | 0.90 (0.67 to 1.20) | 4.70E-01 |  | 0.93 (0.57 to 1.51) | 7.70E-01 |
| Diabetes (type 2) | 1.48 (1.02 to 2.15) | 4.10E-02 |  | 3.11 (1.80 to 5.38) | 5.00E-05 |
| Chronic kidney disease | 0.86 (0.46 to 1.59) | 6.30E-01 |  | 0.37 (0.12 to 1.16) | 8.90E-02 |
| Depression | 1.08 (0.74 to 1.59) | 6.80E-01 |  | 1.16 (0.62 to 2.18) | 6.30E-01 |
| Dementia | 2.65 (0.99 to 7.11) | 5.30E-02 |  | 8.39 (2.20 to 32.00) | 1.80E-03 |
| Asthma | 1.34 (0.93 to 1.93) | 1.20E-01 |  | 0.56 (0.28 to 1.12) | 1.00E-01 |
| COPD | 0.71 (0.47 to 1.09) | 1.20E-01 |  | 0.92 (0.47 to 1.79) | 8.10E-01 |
| Osteoporosis | 1.08 (0.58 to 1.99) | 8.10E-01 |  | 0.82 (0.29 to 2.32) | 7.10E-01 |
| Osteoarthritis | 0.94 (0.66 to 1.33) | 7.10E-01 |  | 1.08 (0.61 to 1.90) | 7.90E-01 |
| **Previous disease/condition*** |  |  |  |  |  |
| Delirium | 0.24 (0.07 to 0.78) | 1.80E-02 |  | 0.15 (0.02 to 1.04) | 5.50E-02 |
| Pneumonia | 0.84 (0.55 to 1.27) | 4.00E-01 |  | 0.88 (0.44 to 1.75) | 7.20E-01 |
| Falls/Fragility fractures | 0.80 (0.59 to 1.07) | 1.30E-01 |  | 0.78 (0.48 to 1.25) | 2.90E-01 |

COVID-19 positive (n=507) compared to COVID-19 negative (n=596). COVID-19 positive and dead (n=141) compared to CVID-19 negative (n=596). Full model (adjusted for age group, sex, ethnicity, education, baseline assessment centre and all the above diseases/conditions).

*Diagnoses from baseline self-report and hospital inpatient admissions

**Comparison group excluded participants testing positive and surviving.
